# Recycling of zinc ions in disc-donut column considering forward mixing mass transfer, and effects of pulsed and non-pulsed condition

**DOI:** 10.1038/s41598-022-05710-0

**Published:** 2022-01-31

**Authors:** Mehdi Asadollahzadeh, Rezvan Torkaman, Meisam Torab-Mostaedi, Mojtaba Saremi

**Affiliations:** 1 Nuclear Fuel Cycle Research School, Nuclear Science and Technology Research Institute, P.O. Box: 11365-8486, Tehran, Iran; 2 Energy Engineering and Physics Department, Amirkabir University of Technology, P.O. Box: 15875-4413, Tehran, Iran

**Keywords:** Engineering, Mathematics and computing

## Abstract

The current study focuses on the recovery of zinc ions by solvent extraction in the pulsed contactor. The Zn(II) ions from chloride solution were extracted into the organic phase containing di-(2-ethylhexyl) phosphoric acid (D2EHPA) extractant. The resulting data were characterized for the relative amount of (a) pulsed and no-pulsed condition; and (b) flow rate of both phases. Based on the mass balance equations for the column performance description, numerical computations of mass transfer in a disc-donut column were conducted and validated the experimental data for zinc extraction. Four different models, such as plug flow, backflow, axial dispersion, and forward mixing were evaluated in this study. The results showed that the intensification of the process with the pulsed condition increased and achieved higher mass transfer rates. The forward mixing model findings based on the curve fitting approach validated well with the experimental data. The results showed that an increase in pulsation intensity, as well as the phase flow rates, have a positive impact on the performance of the extractor. In contrast, the enhancement of flow rate led to the reduction of the described model parameters for the adverse phase.

## Introduction

The find of many applications in various extraction processes such as chemical, and petroleum industries is one of the main advantages of pulsed solvent extraction columns^1,2^. The main specific characteristic of this column is the utilization of pulsing conditions for the intensified process; it is possible not only to increase its efficiency; but also to obtain compactness of equipment, to diminish maintenance efforts, and to improve the quality of extracted products^3–5^. However, these columns have grown well in the extraction stages from a primary source such as the leaching aqueous solution^6–9^. But, its path to secondary resource development and recovery has slowed in industries. Recovery of toxic metals from wastewaters is one of the needs of environmental refinement done by various processes^10–14^. The use of the solvent extraction process has expanded with environmentally friendly solvents^15,16^. But the solvent type alone is not enough to achieve the desirability, and the selected equipment must have a good performance^17^.

Zinc is one of the heavy metals with adverse effects on water resources, soils, vegetables, and crops^18^. In some areas, this pollution is hazardous to human health^19^. The extraction and separation of which is critical from an environmental point of view. Jafari and co-workers’ studies showed that zinc could be extracted from a synthetic solution with a high extraction percentage ~ 94% with D2EHPA extractant^20^. The zinc recovery process from effluents such as spent chloride brass pickle liquors^21^, and zinc-plating mud^22^ has been investigated in the literature. Extraction species depend on different acidic conditions and type of extractant (D2EHPA, Bis(2,4,4-trimethylpentyl) dithiophosphinic Acid (Cyanex301), bis/2,4,4-trimethylpentyl/ phosphinic acid (Cyanex272), 5-nonylacetophenona oxime (LIX 84IC)), and ionic liquids. The zinc recovery from the complex ore resources was investigated by a mixture of trioctylphosphine oxide (TOPO) and tricaprylylmethylammonium chloride (Aliquate336) in the extraction stage and the ammoniacal chloride solution in the stripping stage.

Zinc metal extraction depends on various factors such as the concentration of the extractant, the acidity of the solution and other impurities in the solution. Therefore, extraction is limited by the presence of contaminants. Still, the distribution coefficients of zinc in the phosphoric extractant are high and it is transferred to the organic phase with a higher distribution ratio than other ions such as cobalt and manganese. Also, the operating conditions in the extraction columns is very important. The higher extraction efficiency is carried out in a column with proper mixing of droplets in continuous phase under agitation or pulsation condition. This mixing creates an interfacial area which is the main factor in the mass transfer between the two phases.

The synergistic effects in the extraction stage and the antagonistic effects in the stripping stage are the advantages of using a mixture of extractants^23^. The melting effluents are rich sources of heavy metals such as zinc, copper, nickel, and cadmium. Studies on the recovery of these ions with 2-hydroxy-5-nonylacetophenone oxime and Cyanex272 have shown that the solvent extraction process can play an influential role in their recovery and reduce environmental impact^24^. The technology for extracting zinc from molten effluents has been extensively developed, but the issue of removing other ions has received little research. Song and co-workers reported the extraction of cobalt ions from these effluents for recovery due to the value of cobalt in various industries^25^.

In addition to experimental work, it is possible to describe the process based on different models and mass transfer coefficients^26–30^. The mass balance induced by rising droplets or continuous phase can be crucial in the numerical modeling of the extraction column^31^. Using the mathematical models of the extraction process, it is possible to deduce the dependences of the mass transfer coefficient on the operating condition, and the dependence of the mass transfer coefficient on the physical properties^32,33^. The evaluation of concentration profile curves is of paramount importance, especially for establishing the optimum operating conditions, determining parameters used for scale-up and process design. Mathematical modeling of the extraction column is fundamental due to the economic potential^34^. It is important to develop models for the process to optimize the extraction operation for commercial applications. However, such predictions require establishing a model predicting column performance^35,36^. The plug flow model (PFM) is common with simple assumptions in determining mass transfer coefficients. The backflow model (BFM) and axial dispersion model (ADM) are described by determining the specific coefficients for the deviation from the ideal state^31^. The forward mixing model (FMM) is more superior to the three mentioned models. Applying droplet size distribution in the model helps bring the extraction conditions closer to the actual conditions^37,38^. The exchange of mass transfer between the aqueous and organic phases takes place in the interface of two phases. Interfacial tension significantly affects the droplet size distribution and the required zone for mass transfer. Therefore, the interface of both phases and the creation of different mass transfer phenomena between droplets are very effective in increasing the mass transfer coefficients. Also, interfacial energy dominates over the volume force with considerable surface to volume ratio in micro scale. This phenomenon leads to distinct behavior from those of the macroscopic systems^39^.

**Figure 1:**
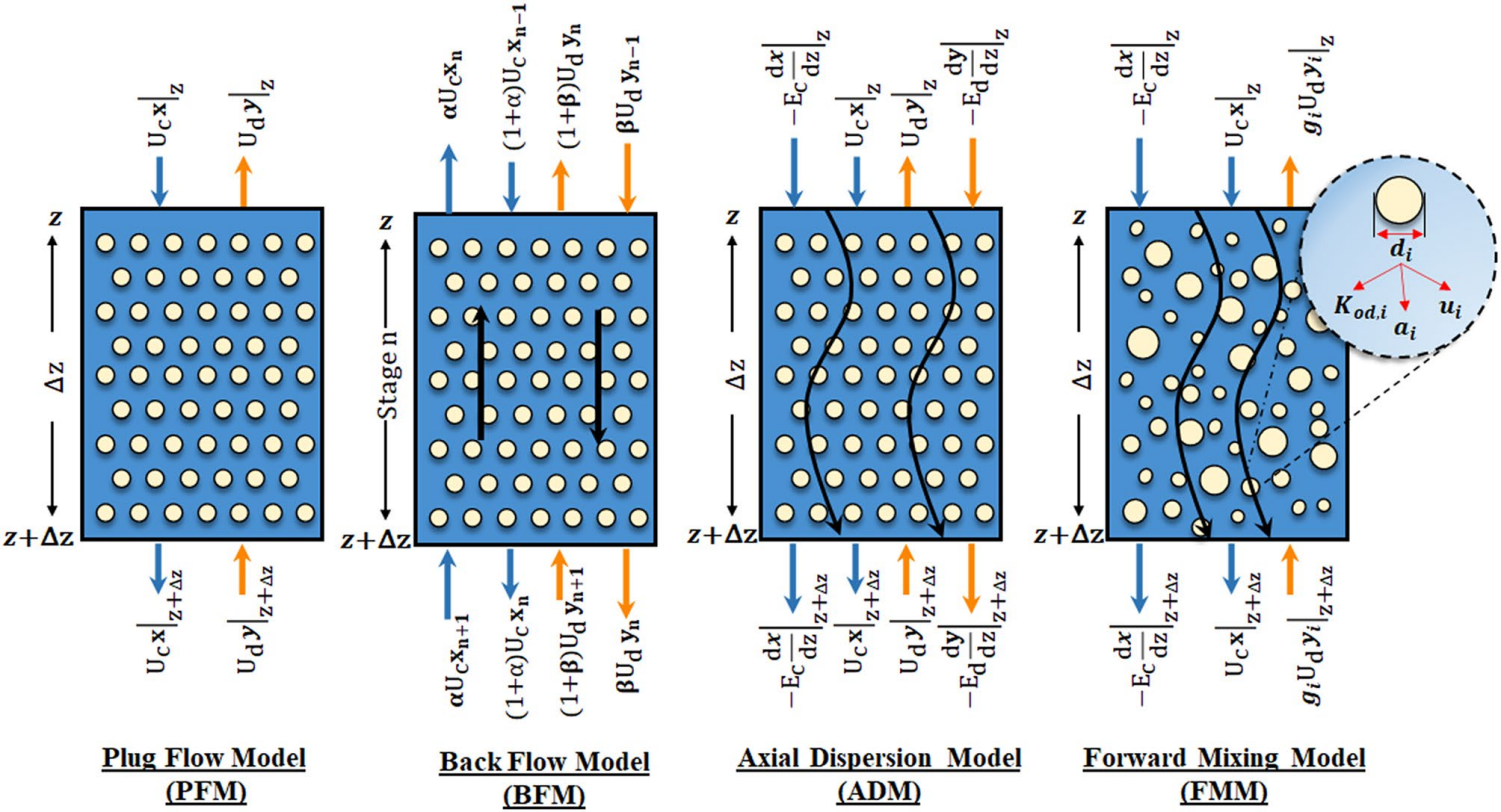
Mass balance over a volumetric element based on the plug flow, back-flow, axial dispersion and forward mixing models.

Mass balance equations illustrate these models, these equations are described in Table 1, and a schematic of the mass transfer equilibrium based on the PFM, BFM, ADM, and FMM models is given in Fig. 1.

**Table 1:** Mass balance equations for four models^31^.

Model	Governing equations	Remarks	Assumptions
PFM	$\frac{dX}{{dZ}} + {\Omega }NTU_{od} \left( {X - Y} \right) = 0$	Continuous phase mass balance	The assumptions of this model are pure plug flow, no axial mixing, equal diameter, velocity, residence time and mass transfer rate for all droplets, the constant of mass transfer coefficient for all droplets, no breakage and coalescence of drops
	$\frac{dY}{{dZ}} + NTU_{od} \left( {X - Y} \right) = 0$	Dispersed phase mass balance	
	$Z = 0 \to X^{0} = X^{in} = 1$	Boundary Conditions at top of column (z = 0)	
	$Z = 1 \to Y^{1} = Y^{in} = 0$	Boundary Conditions at bottem of column (z = H)	
BFM	$\left( {1 + \alpha } \right) X_{n - 1} - \left( {1 + 2\alpha } \right) X_{n} + \alpha X_{n + 1} - \frac{{ {\Omega }NTU_{od} }}{N}\left( {X_{n} - Y_{n} } \right) = 0$	Continuous phase mass balance	The assumptions of this model are pure plug flow with axial mixing, an axial mixing with backflow coefficients of α and β, equal values for diameter, velocity, residence time and mass transfer rate of droplets, no breakage and coalescence of drops
	$\left( {1 + \beta } \right) Y_{n + 1} - \left( {1 + 2\beta } \right) Y_{n} + \beta Y_{n - 1} + \frac{{NTU_{od} }}{N} \left( {X_{n} - Y_{n} } \right) = 0$	Dispersed phase mass balance	
	$$Z = 0 \to \left\{ {\begin{array}{*{20}c} {X_{0} + \alpha \left( {X_{0} - X_{1} } \right) = 1} \\ {Y^{0} = Y_{0} = Y_{1} } \\ \end{array} } \right.$$	Boundary Conditions at top of column (z = 0)	
	$$Z = 1 \to \left\{ {\begin{array}{*{20}c} {X^{N + 1} = X_{N + 1} = X_{N} } \\ {Y_{N + 1} - \beta \left( {Y_{N} - Y_{N + 1} } \right) = 0} \\ \end{array} } \right.$$	Boundary Conditions at bottem of column (z = H)	
ADM	$\frac{dX}{{dZ}} - \frac{1}{{Pe_{c} }}\frac{{d^{2} X}}{{dZ^{2} }} + {\Omega }NTU_{od} \left( {X - Y} \right) = 0$	Continuous phase mass balance	The assumptions of this model are pure plug flow with axial mixing, a diffusion process with the constant diffusion coefficients of E_c_ and E_d_, equal values for diameter, velocity, residence time and mass transfer rate of droplets, no breakage and coalescence of drops
	$\frac{dY}{{dZ}} + \frac{1}{{Pe_{d} }} \frac{{d^{2} Y}}{{dZ^{2} }} + NTU_{od} \left( {X - Y} \right) = 0$	Dispersed phase mass balance	
	$$Z = 0 \to \left\{ {\begin{array}{*{20}c} {\left( {\frac{{U_{c} }}{{E_{c} }}} \right) \left( {1 - X^{0} } \right) = - \left. {\frac{dX}{{dZ}}} \right|_{0} } \\ {\left. {\frac{dY}{{dZ}}} \right|_{0} = 0 \to Y^{0} = Y^{out} } \\ \end{array} } \right.$$	Boundary Conditions at top of column (z = 0)	
	$$Z = 1 \to \left\{ {\begin{array}{*{20}c} {\left. {\frac{dX}{{dZ}}} \right|_{1} = 0 \to X^{1} = X^{out} } \\ {\left( {\frac{{U_{d} }}{{E_{d} }}} \right) \left( {Y^{1} } \right) = - \left. {\frac{dX}{{dZ}}} \right|_{1} } \\ \end{array} } \right.$$	Boundary Conditions at bottem of column (z = H)	
FMM	$\frac{dX}{{dZ}} - \frac{1}{{Pe_{c} }} \frac{{d^{2} X}}{{dZ^{2} }} + {\Omega }\mathop \sum \limits_{i = 1}^{N} NTU_{od,i} \left( {X - Y_{i} } \right) = 0$	Continuous phase mass balance	The main assumptions of this model are the constant values of inlet and outlet flow rates, the use of the axial dispersion coefficient (Ec) for the deviation of the continuous phase from the plug state, no coalescence and breakage of dispersed phase droplets
	$\frac{{dY_{i} }}{dZ} + \frac{{NTU_{od,i} }}{{g_{i} }} \left( {X - Y_{i} } \right) = 0 \left( {i = 1, 2, \ldots N} \right)$	Dispersed phase mass balance	
	$$Z = 0 \to \left\{ { \begin{array}{*{20}c} {\left( {\frac{{U_{c} }}{{E_{c} }}} \right) \left( {1 - X^{0} } \right) = - \left. {\frac{dX}{{dZ}}} \right|_{0} } \\ \\ { Y_{i}^{0} = Y_{i}^{out} \left( {i = 1, 2, \ldots N} \right) } \\ \end{array} } \right.$$	Boundary Conditions at top of column (z = 0)	
	$$Z = 1 \to \left\{ {\begin{array}{*{20}c} {\left. {\frac{dX}{{dZ}}} \right|_{1} = 0 \to X^{1} = X^{out} } \\ {\begin{array}{*{20}c} \\ {Y_{i}^{1} = Y^{in} = 0 \left( {i = 1, 2, \ldots N} \right)} \\ \end{array} } \\ \end{array} } \right.$$	Boundary Conditions at bottem of column (z = H)	
	$g_{i} = \frac{{f_{i} u_{i} }}{{\mathop \sum \nolimits_{j = 1}^{N} f_{j} u_{j} }}$	Dynamic drop size distribution	
	$u_{i} = \frac{{d_{i} }}{{d_{43} }} U_{slip} - \frac{{U_{c} }}{1 - \phi }$	Drop velocity	
	$$d_{43} = \mathop \sum \limits_{i = 1}^{N} \frac{{n_{i} d_{i}^{4} }}{{\mathop \sum \nolimits_{j = 1}^{N} n_{j} d_{j}^{3} }}$$		
	$f_{i} = \frac{{P_{i}^{3} }}{{\mathop \sum \nolimits_{i = 1}^{N} P_{i}^{3} }}$	Volumetric drop size distribution	
	$a_{i} = \frac{{6 \phi_{i} }}{{d_{i} }}$	Drop specific surface area	
	$\phi_{i} = \phi f_{i}$	Drop holdup

Several physical systems have been investigated in the pilot-scale pulsed disc-donut column in the literature^40–43^, but limited studies have been reported with this column to extract metal ions associated with the reaction condition^4,44–46^. One of the new findings and innovations of this paper is the study of different mass transfer models in the extraction of zinc ions. So far, no studies have been reported in this field, and this finding is significant in the design of this pulsed column according to the standard chemical system for zinc extraction.

## Experimental

### Materials

A series of continuous experiments were carried out to remove zinc ions from an aqueous solution at room temperature (25 ± 1.5 °C). The extraction process was carried out by preparing two aqueous and organic solutions. The organic solution was obtained from the dissolution of an extractant from Aldrich company, di-(2-ethylhexyl) phosphoric acid/ D2EHPA (0.1 M) in kerosene. The aqueous solution was obtained from the dissolution of zinc chloride salt (Merck Company, with 99% purity) in distilled water with a concentration equivalent to 600 ppm at pH ~ 6. The details of physical properties are shown in Table 2.

**Table 2:** Characteristics of chemical system used in the pilot-plant column.

Phases		Solute	ρ_c_ (kg/m^3^)	ρ_d_ (kg/m^3^)	μ_c_ (10^−3^ kg/m s)	μ_d_ (10^−3^ kg/m s)	Σ (10^−3^ N/m)
continuous	Dispersed						
Aqueous nitrate solution of Zn(II)	D2EHPA extractant diluted in kerosene	Zinc ions	1011	824	0.975	1.615	18.7

### Pilot plant column

**Figure 2:**
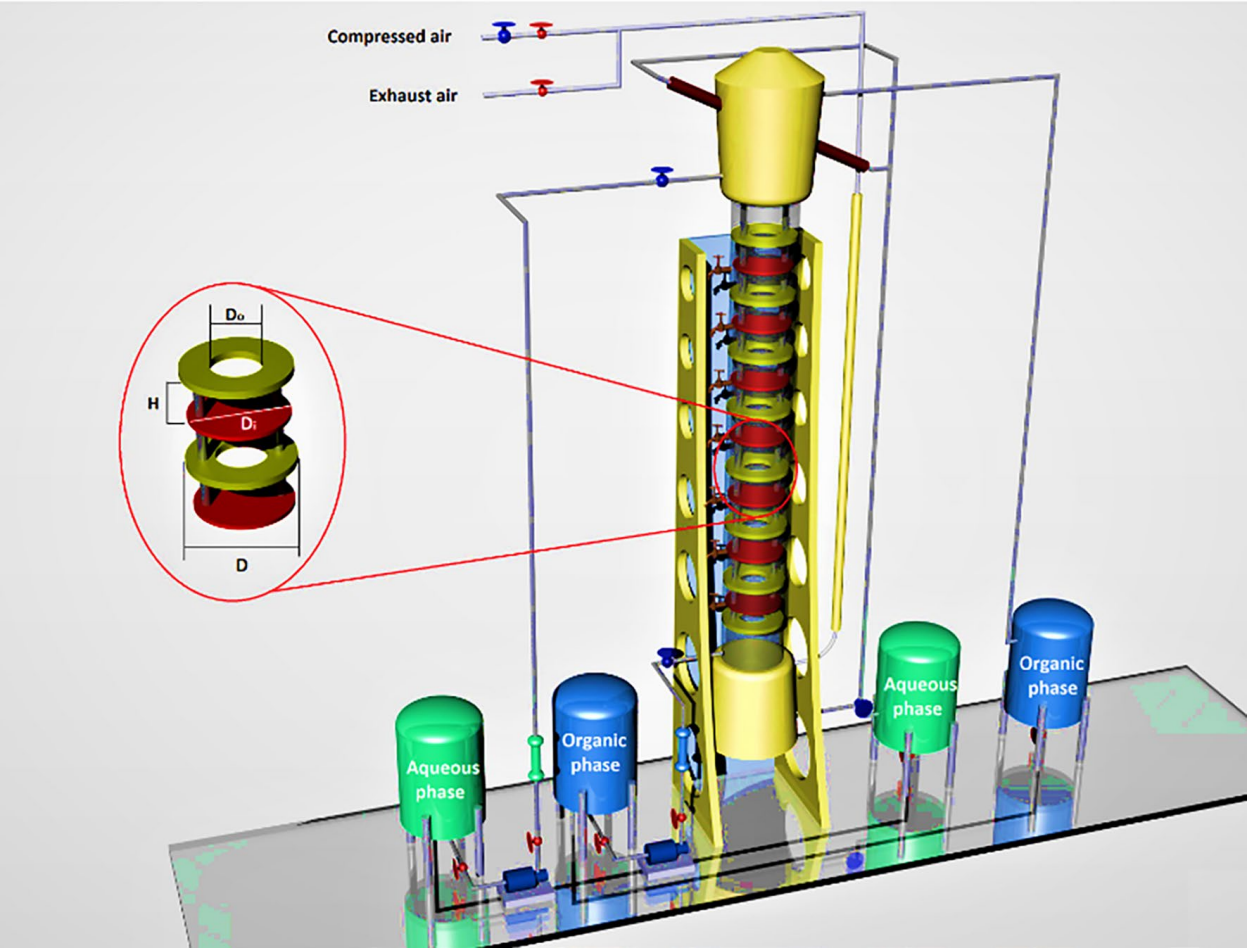
Schematic view of a pilot plant pulsed disc-donut column.

As shown in Fig. 2, the experiments have been carried out in a 0.076 m diameter disc-donut column with a 0.74 m long glass extraction section. The average fractional free area has been maintained at 23.5%. The arrangement of discs and donuts inside the column was made so that 30 discs (0.067 cm O.D) and donuts (0.036 cm I.D) were placed one by one inside the active part.

The aqueous and organic phases are pumped into the column from two stainless steel tanks and the control of flows with two rotameters. The photos of droplets in the column were recorded by a video camera, installed at the center of the column section. The evaluation of drop sizes was obtained by the Sauter mean drop size (d_32_) values for each run. The composition of the aqueous solution in the feed and residual was determined by using UV-spectrophotometer (UNICO model). The shutdown method was used to measure the value of holdup (φ) from the separation of the organic phase in the interface location. The interfacial area for the reaction between zinc ions and extractants in the organic phase was obtained by the following equation:

1 $$ a = \frac{6\varphi }{{d_{32} }} $$

The mass transfer coefficient for the organic-side phase was calculated using four models (PFM, BFM, ADM, and FMM models) in Table 1.

## Results and discussion

The zinc extraction process in the pulsed disc-donut column was continuously investigated in two steps. In the first step, the pulse conditions inside this column were not activated, and only the effects of phase flow rates were investigated in this column. In the second step, pulse conditions were applied inside the column. Different intensities of the pulse were investigated, and the variation in the phase flow rates at different pulse intensities was studied in this column. Four different types of models (PFM, BFM, ADM, FMM models) were evaluated in the performance recognition of this column, according to Table 1. The model analysis result showed that the column behavior using the FMM model is closer to the real values. The errors from the experimental data are much less with the FMM model results. The variation in X-valve and Y-value errors with average absolute relative error (AARE) are presented in Table 3, which indicates that the pulsed disc-donut column is more consistent with the forward mixing model (10.74% for X-value, 13.28% for Y-value).

**Table 3:** Comparison of the AARE values between PFM, BFM, ADM, and FMM models.

Conditions	PFM		BFM		ADM		FMM	
	AAREX (%)	AAREY (%)	AAREX (%)	AAREY (%)	AAREX (%)	AAREY (%)	AAREX (%)	AAREY (%)
Without pulsation	10.93	38.63	5.76	22.35	3.92	11.37	2.38	7.87
With pulsation	13.66	22.48	11.44	17.12	12.88	16.37	10.74	13.28

The main reason for reducing errors is related to the distribution of droplets inside the column, which applies the droplet distribution in the forward model and avoids simplifying assumptions that bring the column’s behavior closer to the actual value. Table 3 shows the plug flow model with simple assumptions is associated with more errors. The backflow and axial mixing models are related to significant errors that are acceptable to use in the study of column behavior. The back-mixing and axial dispersion coefficients defined in these models help the system approach the ideal conditions.

**Figure 3:**
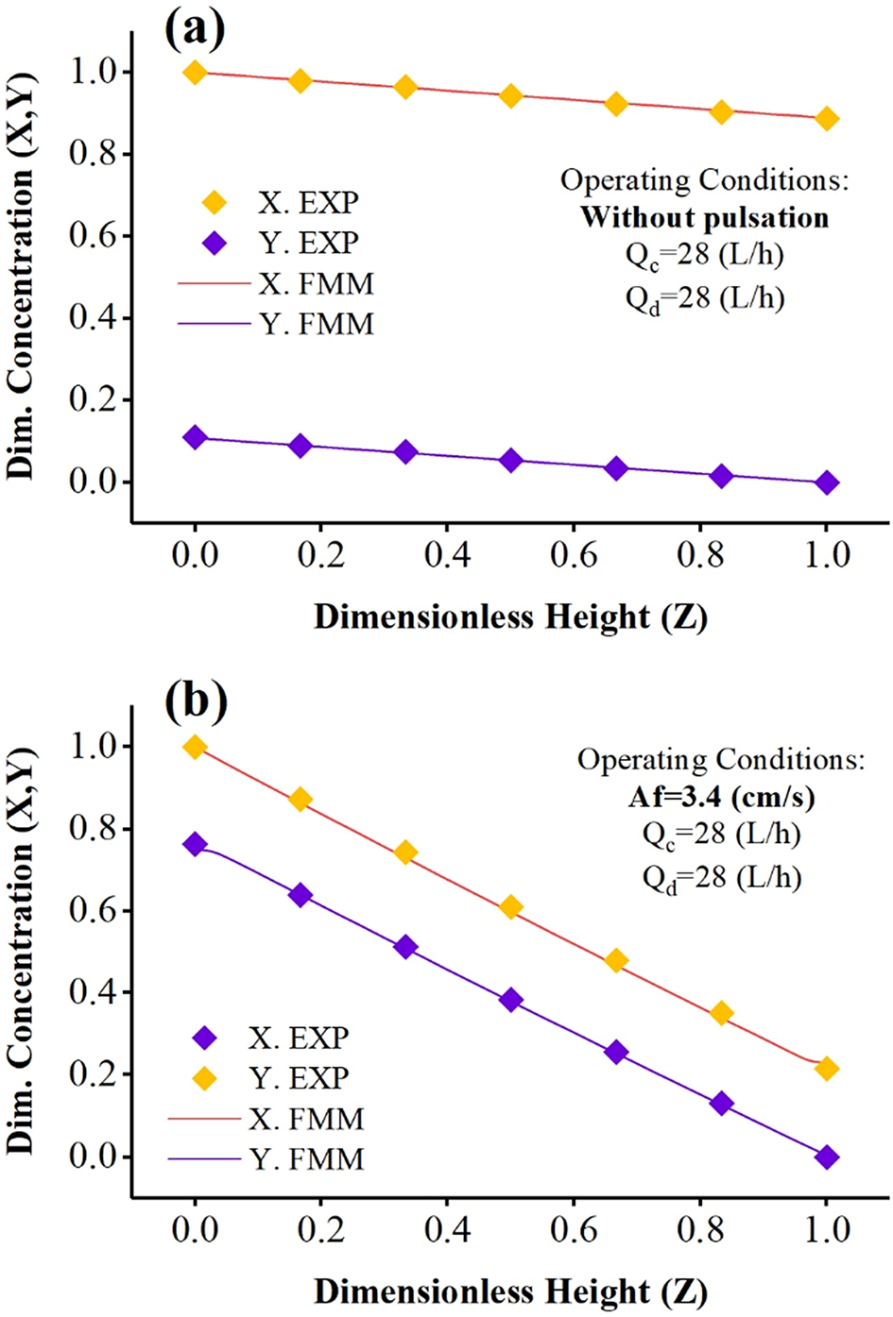
Comparison of laboratory concentration profiles with predicted by the forward mixing model under Q_c_ = Q_d_ = 28 (L/h) and (**a**) without pulsation, (**b**) Af = 3.4 (cm/s).

**Figure 4:**
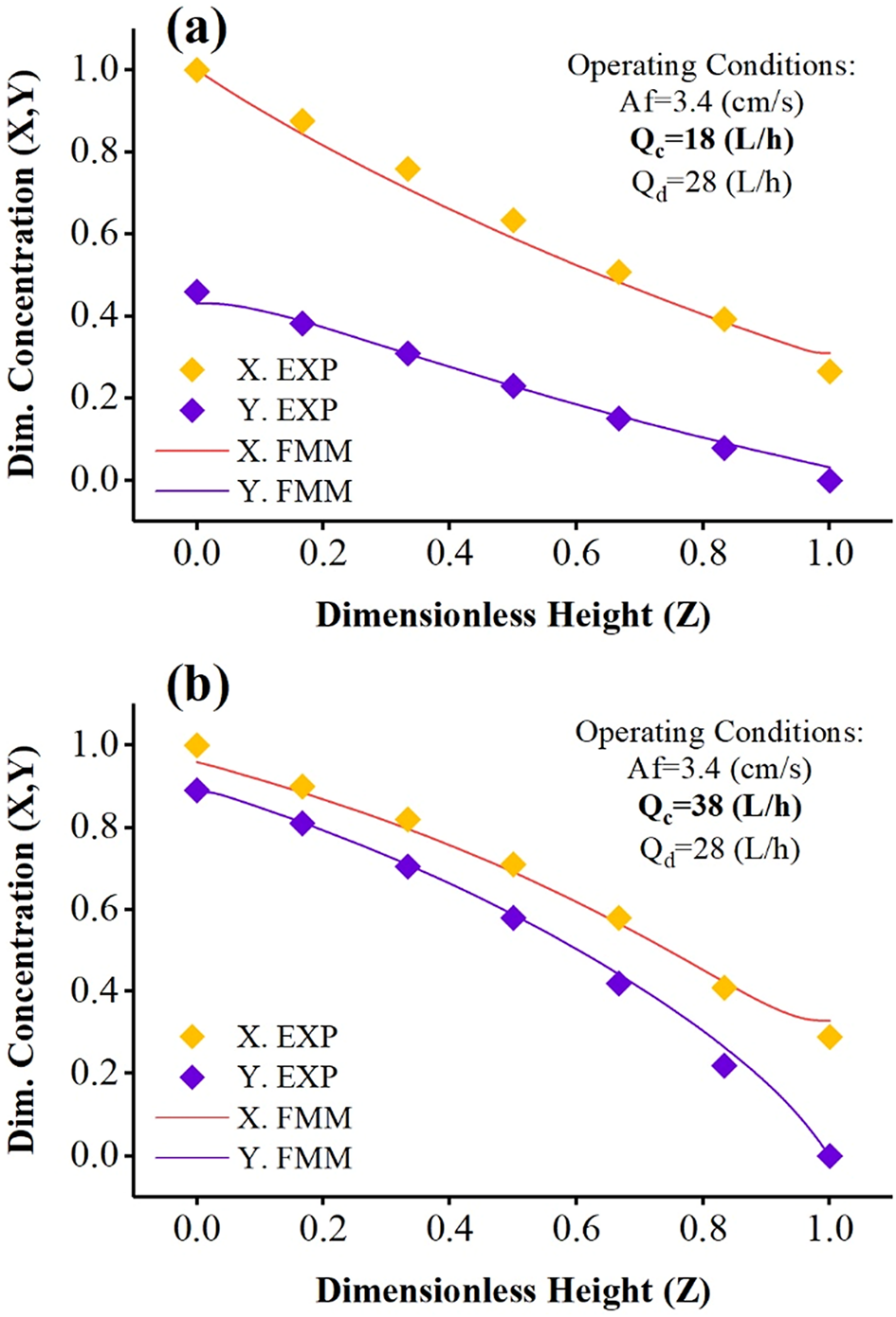
Comparison of laboratory concentration profiles with predicted by the forward mixing model under Q_d_ = 28 (L/h) and Af = 3.4 (cm/s) and (**a**) Q_c_ = 18 and (**b**) Q_c_ = 38 (L/h).

Figures 3 and 4 show the examples of concentration profiles measured by the experimental method under different operating conditions and the concentration profiles predicted by the forward mixing model to extract zinc ions.

The results in this diagram showed that without applying a pulse inside the column, a large mass transfer driving force is established in the column that cannot reach the desired mass transfer and efficiency (see Fig. 3a). But when the pulse is applied inside the column, the column conditions lead to better mass transfer and intensification of the process, which reduces the driving force of the mass transfer is shown in Fig. 3b.

Increasing the continuous phase flow rates in Figs. 3b, 4a, b, increasing the phase flow ratio (Q_c_/Q_d_) causes a change in the mass transfer’s driving force. The decrease in driving force indicates that the phase change curves of the phases are getting closer to each other. The system is closer to ideal conditions and optimal extraction. Therefore, creating pulse conditions and increasing the inlet aqueous phase flow rates intensify the process and reach equilibrium concentrations. Another finding in these two graphs is the correspondence between the forward model and the laboratory data, which shows that the descriptive model can be used with high accuracy in predicting the results.

**Figure 5:**
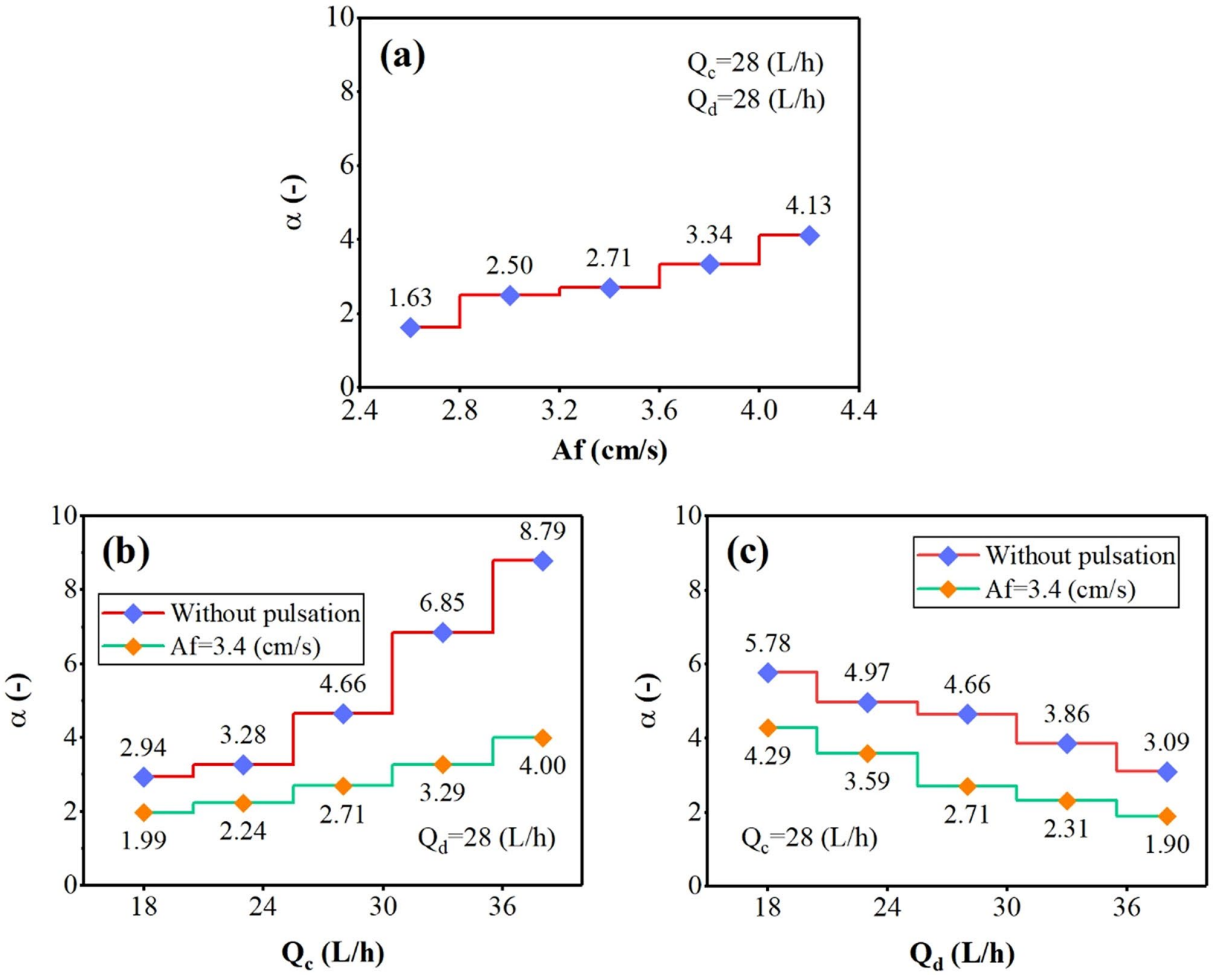
The effect of operating parameters [(**a**) pulsation intensity, (**b**) aqueous phase flow rate, and (**c**) organic phase flow rate] on the continuous phase backflow coefficient.

**Figure 6:**
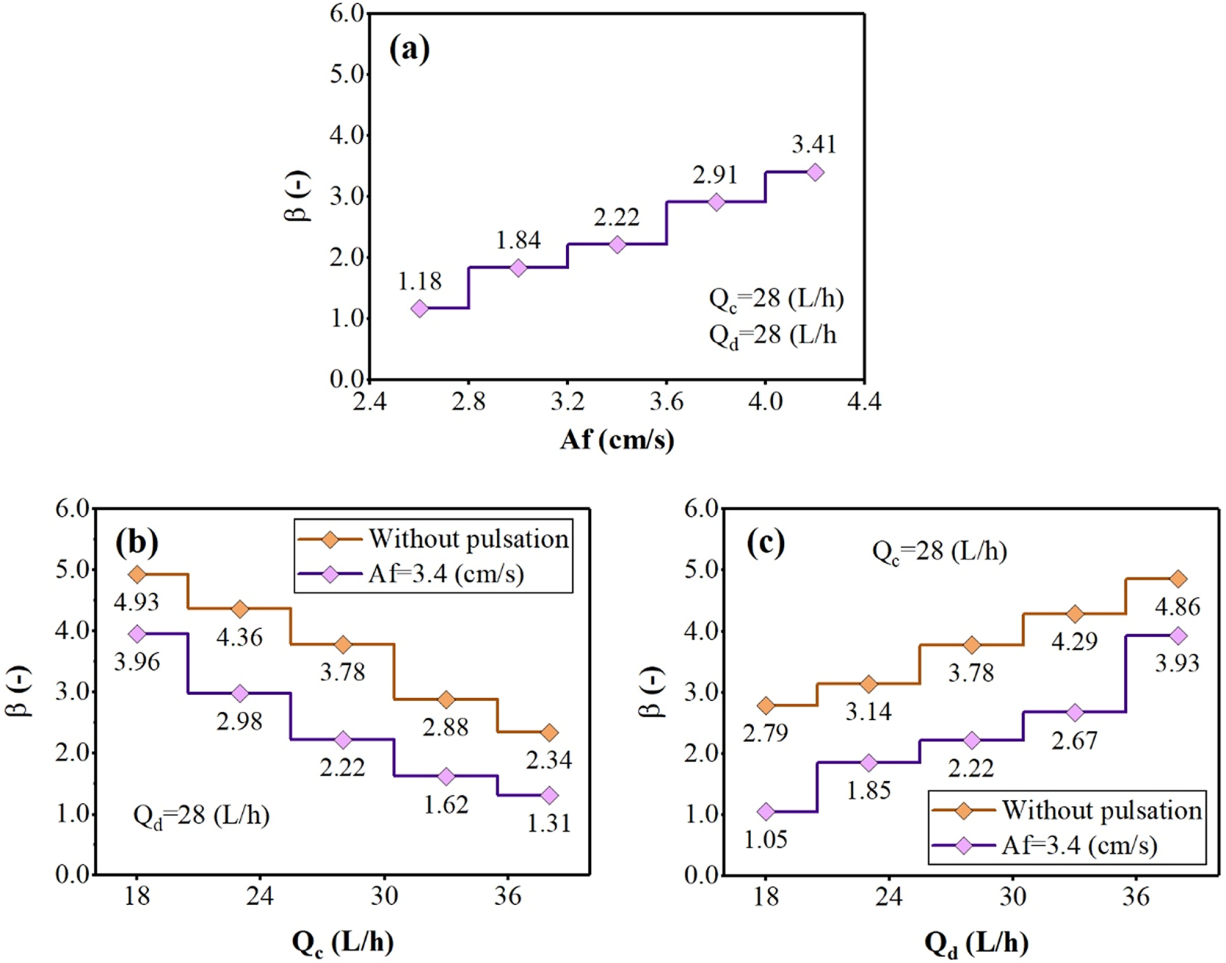
The effect of operating parameters [(**a**) pulsation intensity, (**b**) aqueous phase flow rate, and (**c**) organic phase flow rate] on the dispersed phase backflow coefficient.

The changes in backflow coefficients for aqueous and organic-side phases are shown in Figs. 5, and 6 by examining the effect of operating parameters. These figures showed that increasing the pulse intensity inside the column leads to increasing the back-flow coefficients. Further stress due to the pulse causes the increase in collision frequency of the droplet with the column’s internal components. This factor increases the backflow coefficients (α, and β).

The effect of increasing the continuous phase flow rate on the α, and β coefficients showed that increasing of Q_c_ increases the frequency of collisions with the internal parts, increasing the aqueous-side phase coefficients (α). But it adversely affects on the organic side-phase coefficients (β), because the higher continuous flow rate helps carry droplets. Increasing the dispersed phase flow rate also reduces α and increases β coefficients. Because with the presence of more droplets inside the column, the coalescence rate increases, which has the opposite effect on the α coefficient, but also helps increase the number of collisions at a constant volume for a higher number of droplets and increase the β coefficient.

The results showed that the lack of pulse inside the column is associated with an increase in these coefficients and increases the deflection conditions of the plug flow. However, the use of pulses inside the column causes the necessary stresses to provide a better flow, which intensifies the column’s process and better performance by describing the mass transfer coefficients.

**Figure 7:**
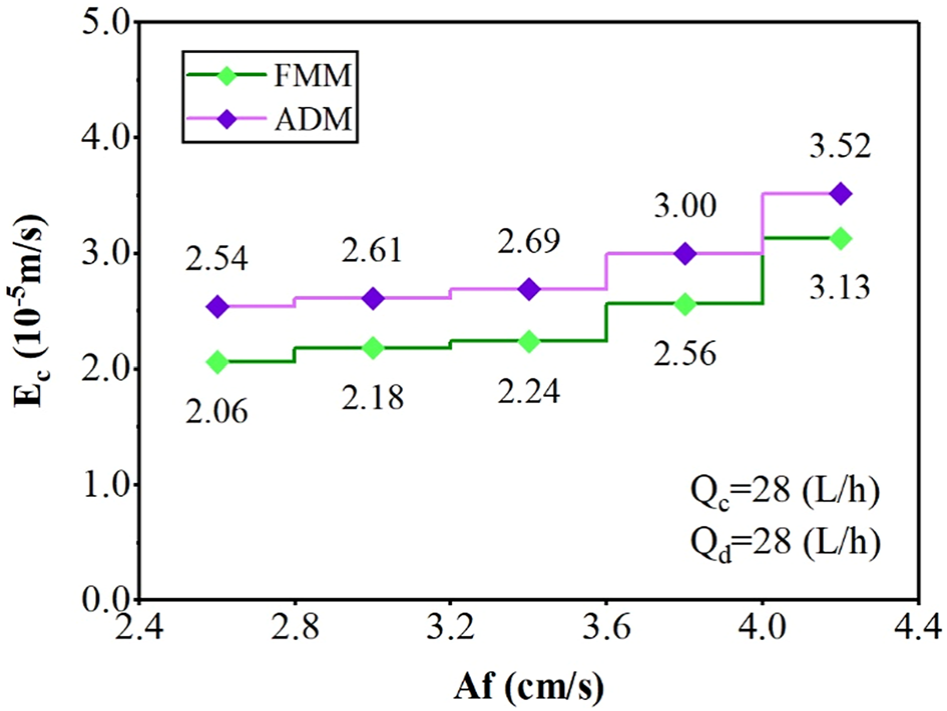
The effect of pulsation intensity on the continuous phase axial dispersion coefficient.

The results in Fig. 7 described that increasing the pulse intensity at constant phase flow rates increases the axial dispersion coefficient for the aqueous side-phase. With increasing turbulence in the system, the droplet aggregation and the vortex formation increase in a continuous phase. Therefore, the higher values for E_c_ are observed with an increment of Af.

**Figure 8:**
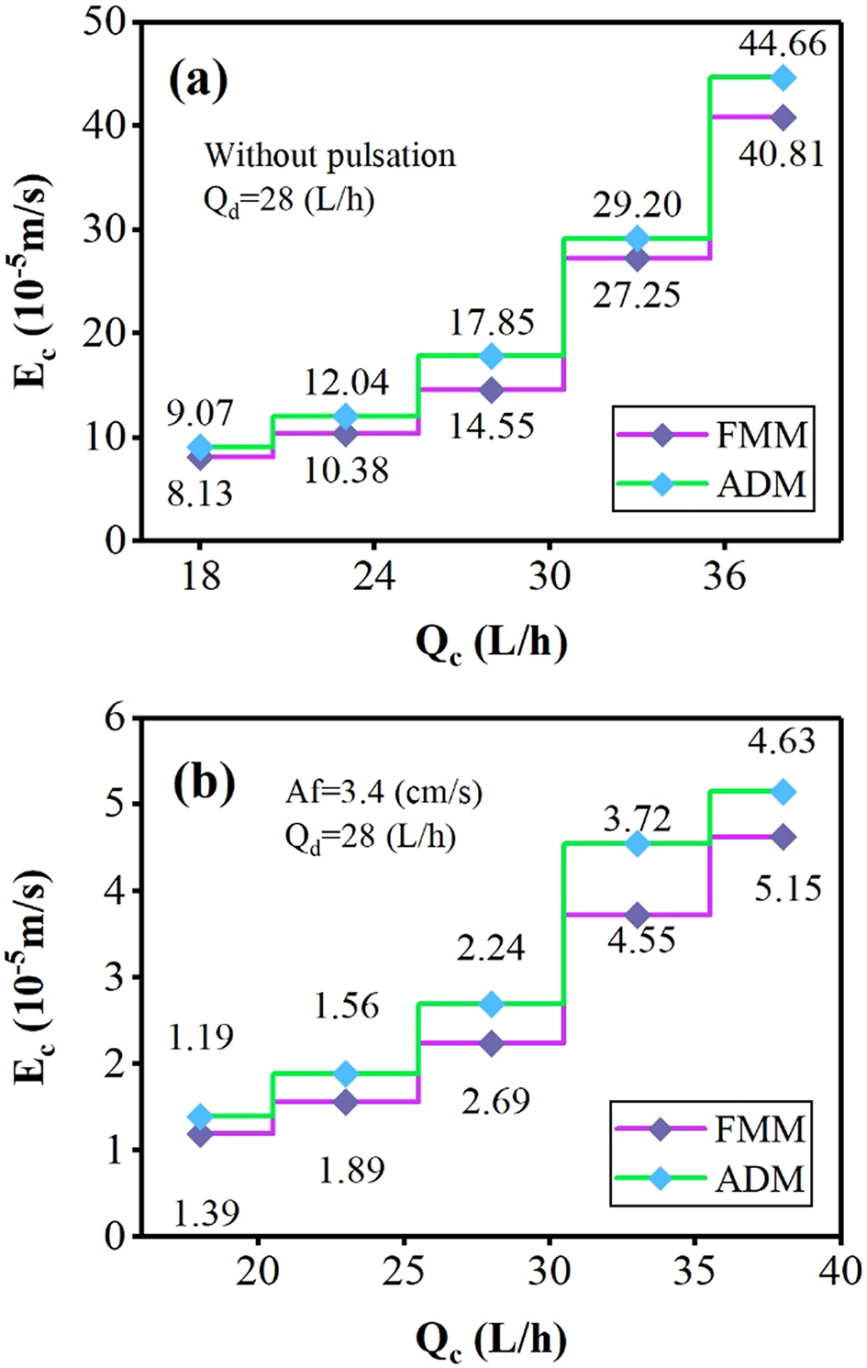
The effect of aqueous phase flow rate on the continuous phase axial dispersion coefficient (**a**) without pulsation, (**b**) Af = 3.4 (cm/s).

**Figure 9:**
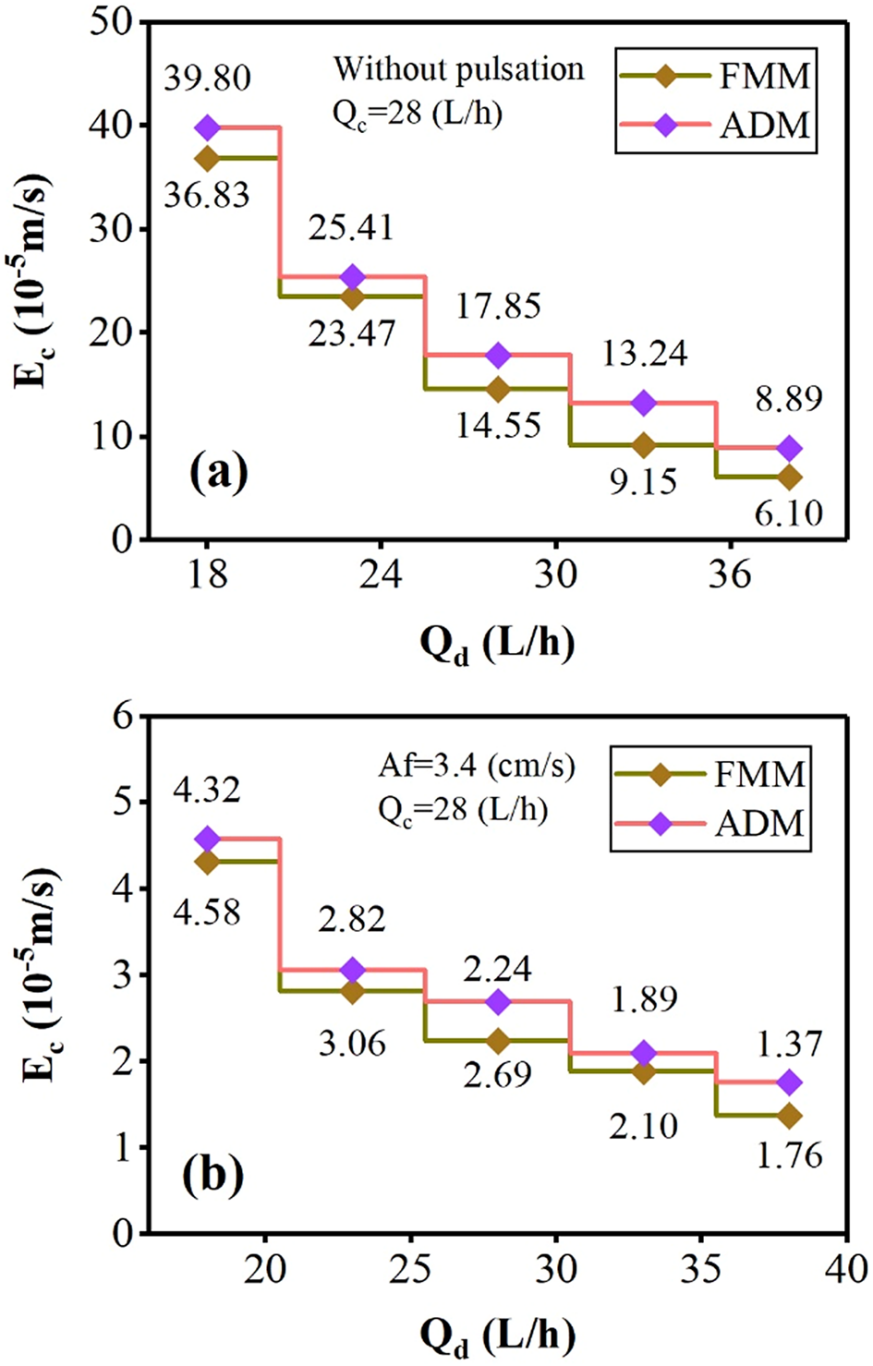
The effect of organic phase flow rate on the continuous phase axial dispersion coefficient (**a**) without pulsation, (**b**) Af = 3.4 (cm/s).

The variations in the dispersed and continuous phase flow on the axial dispersion coefficient of the continuous phase are shown in Figs. 8 and 9. The increase in Q_c_ reveals a positive effect, and the increase in Q_d_ reveals a negative effect on the E_c_. The decrement impact is due to uniform conditions with a reduction in collision frequency of droplets with the internal components. The incremental impact is due to the presence of droplets and participation in more turbulence through the column. These coefficients are much larger in non-pulsed conditions, where the deviations from the ideal current are much larger, and the driving force for mass transfer is high, and reaching the perfect conditions is not achieved by changing the phase flow rate alone.

**Figure 10:**
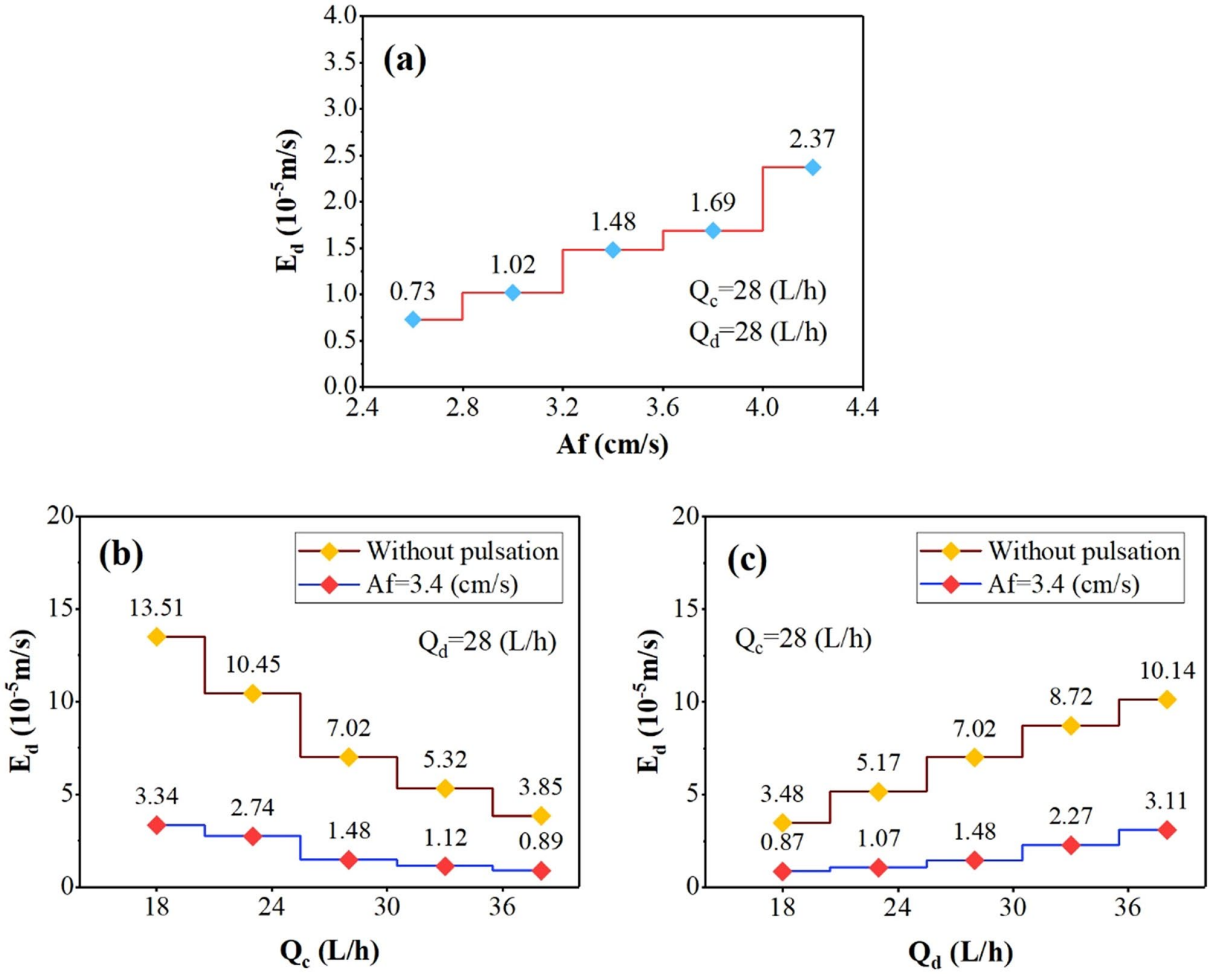
The effect of operating parameters [(**a**) pulsation intensity, (**b**) aqueous phase flow rate, and (**c**) organic phase flow rate] on the dispersed phase axial dispersion coefficient.

The axial dispersion coefficients for the organic-side phase also change similar to continuous axial dispersion coefficients with operating parameters, as shown in Fig. 10. The increase of this parameter is observed by the pulse intensity in Fig. 10a, c. Its decrease is observed by Q_c_ in Fig. 10b. Changes in the droplet’s behavior inside the column under operating parameters help to significantly affect these coefficients, and the variation in flow deflection becomes apparent.

**Figure 11:**
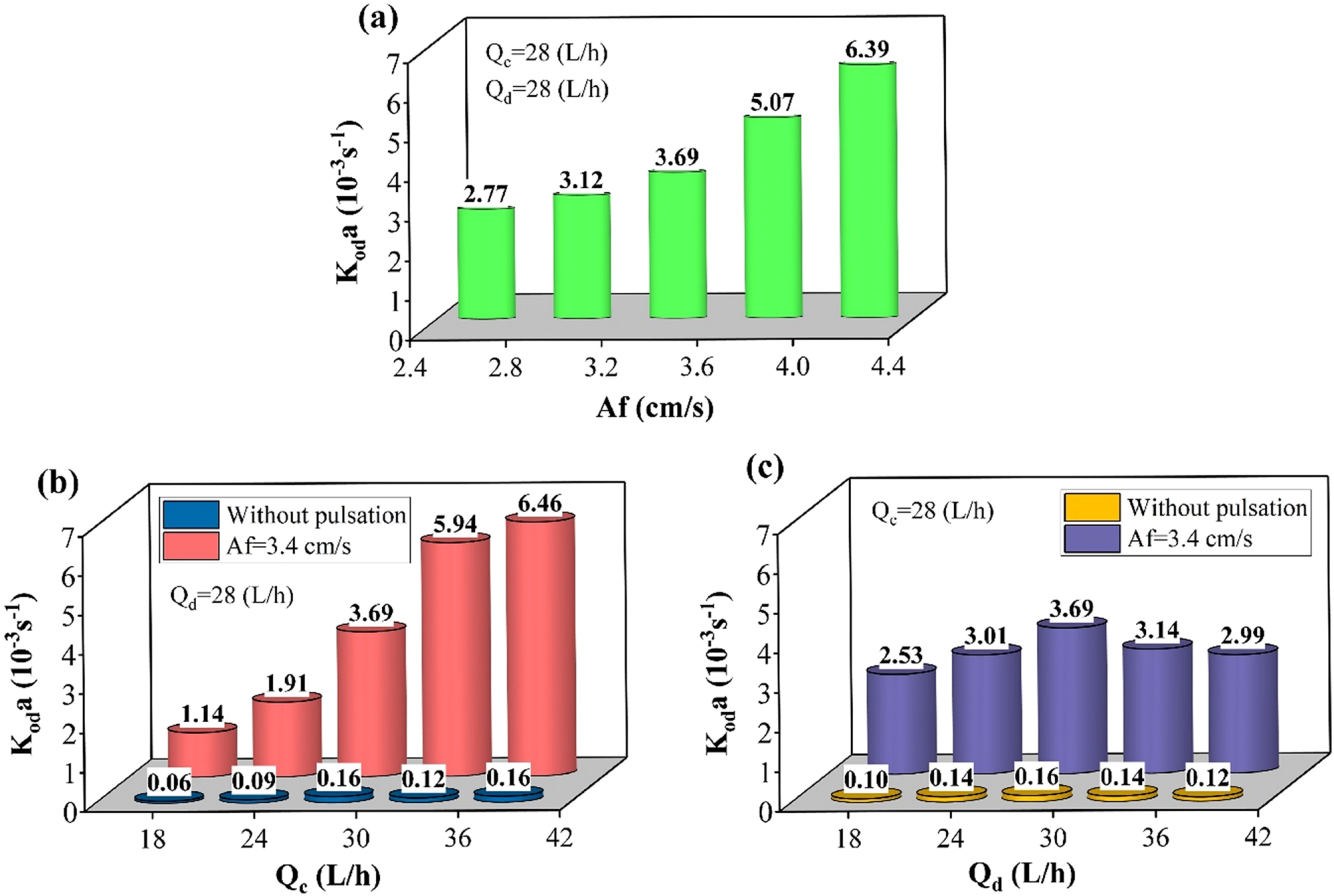
The effect of operating parameters [(**a**) pulsation intensity, (**b**) aqueous phase flow rate, and (**c**) organic phase flow rate] on the volumetric overall mass transfer coefficient predicted by FMM.

Mass transfer coefficients were evaluated by the forward mixing model, the results of which are shown in Fig. 11. The observation indicated that as the pulse intensity increases, the droplet diameter decreases, and the dispersed phase holdup increases, increasing the effective interfacial area for the reaction. The reduction in mass transfer occurs due to the droplet behavior approaching the rigid state and reducing droplet sizes. But the conditions with increasing the required area for the reaction help provide larger volumetric overall mass transfer coefficients. Increasing the phases’ flow rate helps to shift the mass transfer rates within the droplets toward the internal circulation, as larger droplets are formed when the coalescence rate increases. In addition to increasing the required interfacial area for the reaction, the mass transfer coefficients for the organic-side phase increase along this column. The results in these figures showed that the pulse conditions are an essential factor in intensifying the column process and providing the requirements for mass transfer. Without this factor inside the column, very small mass transfer coefficients are obtained, indicating the column’s poor performance in non-pulsed conditions.

**Figure 12:**
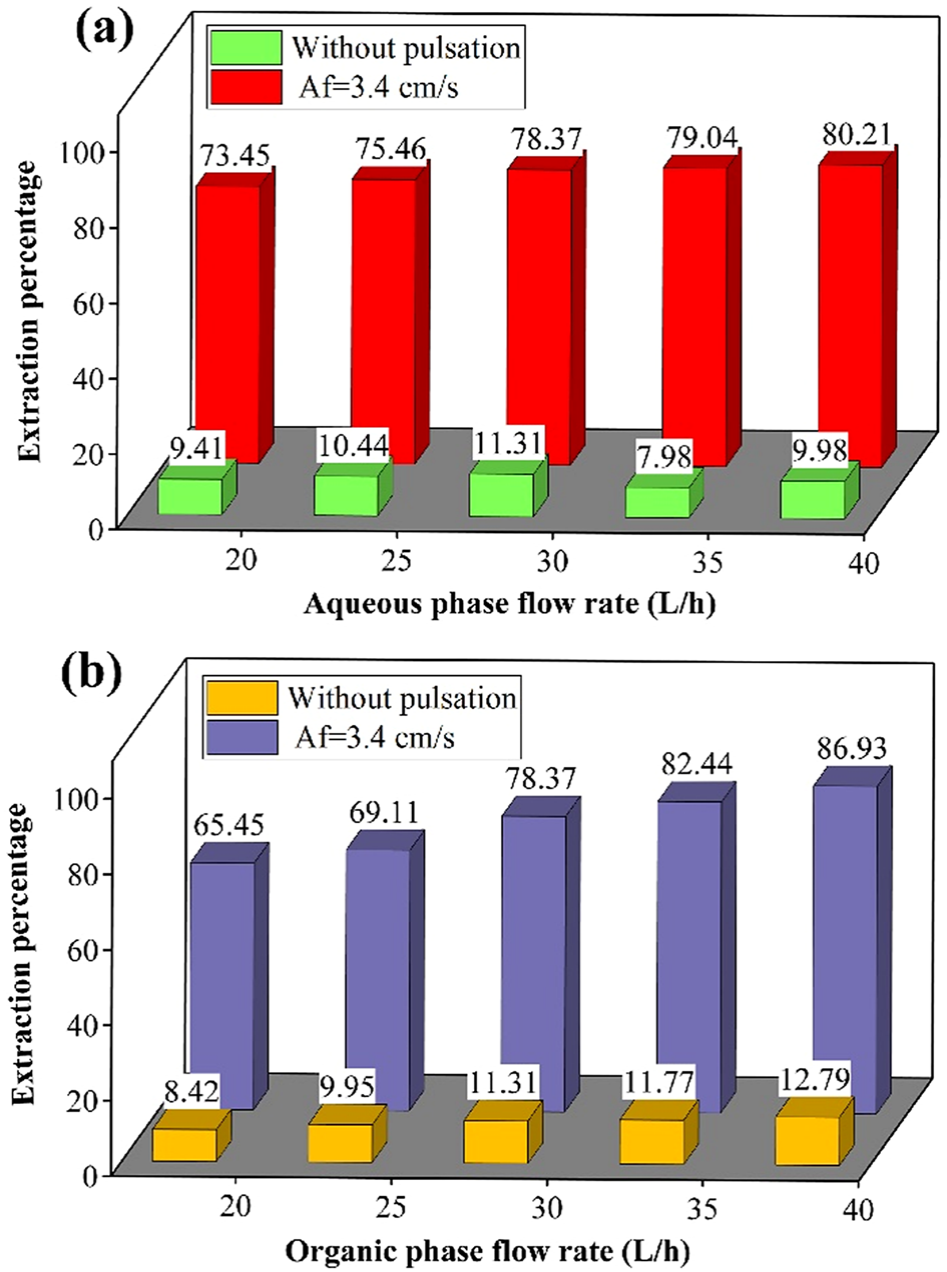
The influence of aqueous and organic phase flow rate on the extraction percentage.

The results of the percentage of zinc extraction with Q_c_ and Q_d_ are shown in Fig. 12. The results illustrated that the zinc ions’ extraction is a function of the inlet phase rates and pulsed conditions. Increasing the flow rate of the phases helps to establish the higher mass transfer rate in the pulsed disc-donut column and the result is an increase in the percentage of extraction of zinc ions from the aqueous to the organic phase. These diagrams also observe the intensification of the process by applying pulses in the column and performing the extraction reaction, and increasing the efficiency.

The results showed that the disc-donut column could be used to remove and recover heavy metals from effluents. The presence of a pulsing condition inside the column provides a higher interfacial area with better mixing, which results in higher mass transfer and higher operating efficiency. Therefore, pulsed disc-donut columns are very desirable and efficient in reaction conditions.

## Conclusion

In this study, the extraction of zinc ions from the aqueous to the organic solution was investigated as a standard reaction system in the pulsed disc-donut column. The operating conditions of the pilot plant column were checked by changing the flow rate of the inlet phases and applying pulses inside the column. The extraction process description was examined the mass transfer models (plug flow, backflow, axial dispersion, and forward mixing models). The results of variation in the operating parameters showed that the optimal extraction conditions are obtained at equal flow rates from the organic and aqueous phases (Q_c_ = Q_d_ = 28 L/h) and the maximum pulse intensity (Af = 4.4 cm/s) in which the volumetric mass transfer coefficient is equal to 0.00639 1/s K_od_ × a, and the extraction efficiency is equivalent to 93.34%. The models’ evaluation showed that the error of the forward mixing model is less than other models in predicting the results. Deformation of the concentration profile inside the column by examining the effect of pulse intensity (Af) and flow rate ratio (Q_c_/Q_d_) showed that reducing in the driving force and achieving equilibrium are the effects of the pulsed condition and the increment in continuous phase flow rate. These parameters help to exchange and transfer more ions between both phases with a higher extraction efficiency.

## List of Symbols

*Af*: Pulse intensity (cm/s)
*a*: Specific surface area per unit volume (6φ/d_32_) (1/m)
*d*: Drop diameter (m or mm)
*d*_32_: Sauter mean drop diameter (mm)
*d*_43_: Mean diameter (m)
*E*: Axial dispersion coefficient (cm^2^/s)
*f*: Volumetric drops size distribution (–)
*g*: Dynamic drop size distribution (–)
*H*: Height of column (m)
*K*: Overall mass transfer coefficient (m/s)
*n*: Number of drops (–)
*N*_*d*_: Number of drop classes (–)
*P*: Drop size distribution
*Q*: Volume flow rate (L/h)
*S*: Cross-section area of the column (m^2^)
*t*: Residence time of drops (s)
*u*: Drop velocity (m/s)
*U*: Superficial velocity (Q/S) (m/s)
*U*_*slip*_: Slip velocity (U_c_/(1 − φ) + U_d_/ φ) (m/s)
*V*: Volume of phases (m^3^)
*x*: Mass fraction of solute in the continuous phase (–)
*y*: Mass fraction of solute in the dispersed phase (–)
*z*: Height variable (m

## Dimensionless symbols

*NTU*: Number of transfer unit (K_od_aH/U_d_)
*p*: Slope of the concentration equilibrium equation (y = p^*^x + q)
*Pe*: Peclet number (HU/E)
*q*: Constant of the concentration equilibrium equation (y = p^*^x + q)
*X*: Continuous phase concentration ((**x** − **x**^*^_out_)/(x_in_ − x^*^_out_))
*Y*: Dispersed phase concentration ((y − y_in_)/(y^*^_out_ − y_in_))
*Z*: Height (Z = z/H)
*Ω*: Constant (pU_d_ρ_d_/U_c_ρ_c_

## Greek symbols

*α*: Continuous phase backflow coefficient (–)
*β*: Dispersed phase backflow coefficient (–)
*μ*: Viscosity (kg/m.s)
*ρ*: Density (kg/m^3^)
*σ*: Interfacial tension between two phases (N/m)
*φ*: Dispersed phase holdup (–

## Subscripts/superscripts

***: Equilibrium concentration
*c*: Continuous phase
*d*: Dispersed phase
*i*: Drop class
*in*: Inlet to the column
*j*: Cross-section
*out*: Outlet from the column
AARE: Average Absolute Relative Error
ADM: Axial Dispersion Model
BFM: Back Flow Model
PFM: Plug Flow Model
FMM: Forward Mixing Model

## Abbreviations

AARE: Average Absolute Relative Error
ADM: Axial Dispersion Model
BFM: Back Flow Model
PFM: Plug Flow Model
FMM: Forward Mixing Model
